# **Brow Ptosis after Upper Blepharoplasty: Findings in 70 Patients**

**Published:** 2016-01

**Authors:** Seyed Esmail Hassanpour, Houman Khajouei Kermani

**Affiliations:** Department of Plastic and Reconstructive Surgery, Shahid Beheshti University of Medical Sciences, Tehran, Iran

**Keywords:** Upper blepharoplasty, Brow ptosis, Complication

## Abstract

**BACKGROUND:**

Brow ptosis is a potential complication after upper eyelid blepharoplasty. The aim of this study was to analyze the effect of upper blepharoplasty on eyebrow position.

**METHODS:**

In this Between April 2011 and March 2013, eighty three patients (166 eyes with mean age of 49.7 years) underwent upper eyelid blepharoplasty. The patients were assessed using pre- and post-operatively digital photographs, in the primary position of the eye while the distance between the upper lid margin and the brow were measured before surgery. The postoperative degree of brow ptosis was evaluated as being mild (<2 mm), moderate (2-4 mm), and marked (>4 mm).

**RESULTS:**

The postoperative brow position was unchanged in 46 cases (65.8%), and brow depression was noted in 24 cases ( 34.2%), including 7 males (58.3%) , and 17 females (29.3%).

**CONCLUSION:**

Our study shows that postoperative brow position should be explained to patients before surgery, particularly in male and senile patients as concomitant brow lift or internal brow fixation through the blepharoplasty incision can help to stabilize the eyebrow in the proper position and to prevent this complication.

## INTRODUCTION

The eyes and periorbital area are the focal point during human communication. Changes in the eyelid appearance due to aging may convey an inappropriate message of sadness, tiredness, and absence of vigor, which may reduce the aesthetic appearance of the face.^1^^,^^2^ So blepheroplasty is a complex and challenging issue that continues to be the most common invasive cosmetic surgical procedure of the face.^3^ Upper eyelid blepharoplasty results in improvement of the natural aging changes and improves the appearance.^4^

Postoperative complications of upper eyelid blepharoplasty range from skin changes to vision–threatening emergencies while some of complications are seen early in the postoperative period including infection, retrobulbar hemorrhage, and eyelid hematoma. Other complications happen later in the postoperative period, such as eyelid malposition, over-and under–resection of skin or orbital fat.^5^^-^^7^ The findings on brow after upper blepharophasty is still controversial.^8^ Some authors insist that brow position remains unchanged after surgery. On the other hand, there is also an opinion that brow position changes after surgery.^9^^,^^10^ The aim of this study was to assess the influence of upper blepharoplasty on brow position and to analyze the effect of major risk factors on eyebrow to move down. 

## MATERIALS AND METHODS

Between April 2011 and March 2013, eighty three patients (166 eyes) underwent upper eyelid blepharoplasty. Thirteen cases were excluded because of incomplete follow up referrals. Finally, 70 patients (140 eyes) were included in this study. There were 58 women and 12 men and the mean age of patients was 49.7 years (48.9 years in females and 53.7 years in males). 

Individuals who had undergone lid surgery had eyebrow, pervious eyebrow surgery while those with eyelid ptosis were excluded. Patient age, sex, indications, complications and changes in brow position after upper blepharoplasty were recorded. Photographic documentation was performed by a professional medical photographer in a standardized manner using a Nikon D 300 camera. Preoperatively, surgical landmarks and planed skin excisions were marked on the patients. All operations were performed through a traditional blepharoplasty incision. Immediately after the surgery, an antibiotic ophthalmic ointment was placed over the skin incision. Ice compresses were used for 48 hours, 20 minutes per hour while awake, following the procedure.

Acetaminophen was routinely prescribed, and showering was permitted the day following the procedure. The hospital stay was one day. Patients were then seen 5–7 days after operation to remove the sutures. Patients, were followed for at least 6 months after surgery to monitor surgical results, so these patients underwent no other procedures, either before or during the time span between the photographs that could affect eyebrow position. 

**Fig. 1:**
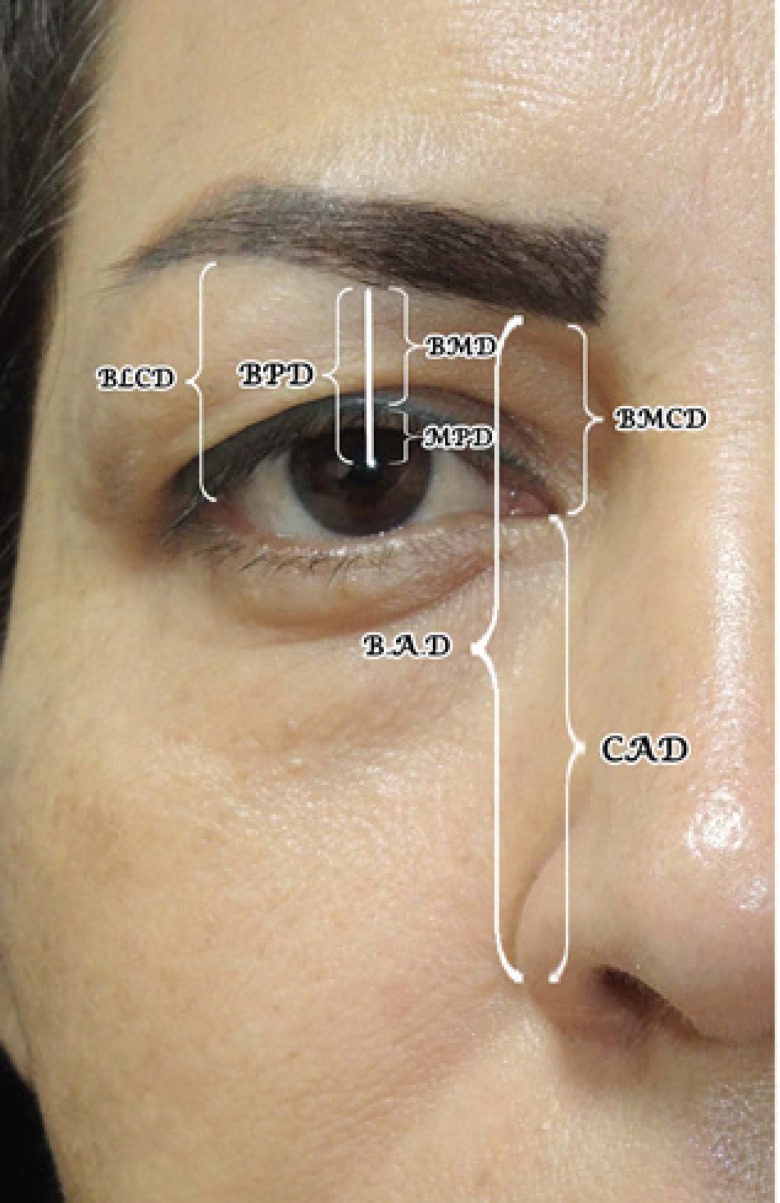
Photography of right eyelid & eyebrow for measurement of parameters

The patients were assessed by means of a comparative analysis using pre– and post–operative digital photographs, in the primary position of the eye. The images were processed using Image J “software”, transferred to a computer, to an electronic Microsoft Excel 2002® worksheet. Distance measurements were used, taking the brow–lid margin distance (BMD), brow-pupil distance (BPD), lid margin- pupil distance (MPD), brow–lateral canthal distance (BLCD), brow–medial canthal distance (BMCD), as anatomical reference points (Figure 1).

BLCD= Brow-lateral canthal distance, BMD= Brow-lid margin distance, MPD= Lid margin-pupil distance, BPD= Brow-pupil distance, BMCD= Brow-medical canthal distance, CAD= Canthal-nasal alar distance, BAD= Brow alar distance

The difference between the pre–operative and 6 months post–operative measurements were analyzed statistically using the student’s t – test for paired samples and the distance variation was compared with their corresponding contralateral sample using Wilcoxon’s non-parametric test. The post-operative degree of brow ptosis was assessed as being mild (<2 mm), moderate (2–4 mm), and marked (>4 mm).

## RESULTS

In all, 140 eyes upper eyelid blepharophasty operations were performed on 70 patients identified from the departmental database. This group of patients consisted of 58 women and 12 men with an average age of 49.7 years (range: 29-68 years). All patients underwent traditional upper eyelid blepharophasty. The mean follow up period was 6 month. The most common chief complaint was dermatochalasis in 57 patients and dermatochalasis with visual field impairment in 13 patients. Echymosis and swelling lasting more than two weeks, were the most frequently documented complications, occurring in 12 cases. There were no complications such as wound infection, wound dehiscence or blindness (Table 1). 

**Table 1:** Demographic features of patients

**Variable**	**Male**	**Female**
Number of patients	12 (17.2%)	58 (82.8%)
Total	70 (140 eyes)	
Age (mean, years, ranges)	53.7 (40-68)	48.9 (29-65)
Overall	49.7 year (29-68)	
Brow depression	7 (58.3%)	17 (29.3%)
Surgical indication	57 (81.4%) cosmetic	
	13 ( 18.6%) cosmetic+visual field defect	

The postoperative degree of brow ptosis was assessed objectively by means of a comparative analysis using pre- and post-operatively digital photographs, in the primary position of the eye, as being mild (<2 mm), moderate (2-4 mm), and marked (>4 mm ) (Table 2).

**Table 2:** Comparisons of objective changes of measured parameters in patients with eyebrow position change

**Variable**	**Mild (<2 mm)**	**Moderate (2-4 mm)**	**Severe (>4 mm)**
Female	11 (64.7%)	4 (23.5%)	2 (11.7%)
Male	4 (57.2%)	2 (28.5%)	1 (14.2%)
Total	15	6	3

Overall: 24 cases (34.2%)

The measurement obtained after the blepharoplasty, showed significant difference from those before the surgery, indicating that the correction of redundant tissues in eyelid accentuates the tendency of the eyebrow to move down. The changes were more significant in the lateral portion of the eyebrow and they occur bilaterally. This study showed that age over 55 years, particularly in male patients may influence brow position after blepharoplasty (Table 3).

**Table 3:** Frequency of brow depression after upper eyelid blepharoplasty

**Age (years)**	**Female **	**Male**
20-29		
30-39		
40-49	2	1
50-59	13	4
>60	2	2
Total	17 (29.3%)	7 (58.3%)

## DISCUSSION

The assessment of distance measurement between the upper lid margin and the brow, obtained pre- and post–operatively, showed that there were secondary changes in the position of the eyebrow as a result of upper eyelid blepharoplasty. Our study also implies to the possibility of a change in postoperative brow position that should be explained to patients before surgery, particularly in male and senile patients. 

Excessive skin in the upper eyelid may be wholly or partially attributable to ptosis of the eyebrows.^11^ In such cases, performing a blepharoplasty alone may inadequately correct the dermatochalasis. More seriously, it may produce further lowering of the eyebrow, that has a compensatory elevated position before blepharophasty leading to an unacceptable cosmetic result.^12^^,^^13^

If the patient had chronic brow strain and elevation before surgery due to the dermatochalasis and the extra skin was removed by performing the blepharoplasty, the brow no longer would need to maintain an elevated position and sank down. If brow strain is evident during preoperative evaluation, strong consideration must be given to brow lift and an upper lid blepharoplasty or ptosis repair is to be performed. 

Preoperative measurements of upper eyelid heights appear useful in determining the amount of skin excision required in blepharoplasty for senile patients.^14^ Therefore, transplapebral techniques should be indicated only with special attention to the age and sex. Concomitant brow lift or internal brow fixation through the blepharoplasty incision can help to stabilize the eyebrow in the proper position and prevention of this complication.^15^^,^^16^

## CONFLICT OF INTEREST

The authors declare no conflict of interest.
